# Integrating the IEEE 1451 and IEC 61499 Standards with the Industrial Internet Reference Architecture

**DOI:** 10.3390/s22041495

**Published:** 2022-02-15

**Authors:** Helbert da Rocha, Reza Abrishambaf, João Pereira, Antonio Espirito Santo

**Affiliations:** 1 Department of Electromechanical Engineering, University of Beira Interior, 6200-001 Covilhã, Portugal; joao.luis.pereira@ubi.pt (J.P.); aes@ubi.pt (A.E.S.); 2 Instituto de Telecomunicações, Delegação da Covilhã, 1049-001 Lisboa, Portugal; 3 Department of Engineering Technology, Miami University, Hamilton, OH 45011, USA; abrishr@miamioh.edu

**Keywords:** interoperability IIoT and I4.0, reference architecture models, CPPS, CPS

## Abstract

Industrial Internet of Things focuses on the manufacturing process and connects with other associated concepts such as Industry 4.0, Cyber-Physical Systems, and Cyber-Physical Production Systems. Because of the complexity of those components, it is necessary to define reference architectures models to manage Industry 4.0 and the Industrial Internet of Things. The reference architecture models aim to solve the interoperability problem enabling the syntactical and semantic levels of interoperability. A reference architecture model provides a bottom/top view of an industrial process, from the physical transducers at the physical layer to the business layer. The physical layer provides access to a twin representation of a physical thing in the digital world, extending the functionalities in the manufacturing process. This paper studies the syntactic interoperability between the IEEE 1451 and IEC 61499 in an industrial environment. The IEEE 1451 family of standards has the essential characteristics to support the information exchange between smart transducers (sensors and actuators), building the digital elements and meeting the Industry 4.0 requirements. The IEC 61499 standard enables industrial control and automation. These two standards combined at the syntactic level solve an interoperability problem. The IEC 61499 also provides data to the framework layer, supplying all the parameters defined for the communication layer specified by a reference architecture model. This paper combines the IEEE 1451 with the IEC 61499, enabling data exchange in a reference architecture model proposed for Industry 4.0. Network performance at the communication level of a reference architecture model in a local network and an external network is evaluated for the proposed application. The IEEE 1451 standard implementation and adoption to acquire data and communicate it inside an industrial process allowed the IEC 61499 standard to control an industrial process. The IEEE 1451 standard is implemented in a MSP430 low power microcontroller. A Raspberry Pi running FORTE and 4diac in the USA and Portugal were used to test a local network in Portugal and an external network in USA. Data related to network performance was obtained with Wireshark and processed with MATLAB. Tests using the Message Queuing Transport Telemetry Transport and Hypertext Transport Protocols verified the performance of these protocols, supported by the IEEE 1451 and IEC 61499 standards, showing that communication inside an Industry 4.0 environment is possible. MQTT protocol is faster, has a small packet size, and consumes less bandwidth. The HTTP protocol uses more bandwidth but is more reliable for real-time communication, essential for Industry 4.0.

## 1. Introduction

The need for a reference architecture appears once new initiatives are under development to work toward a standardization architecture. The reference architecture enables interoperability, simplifies development, and provides a straightforward implementation [1]. A reference architecture is a generic guideline from the requirements and functionalities, relationships, principals, information structure, and mechanisms that do not necessarily propose the specific detail about the actual implementation. The reference architecture focuses on connectivity and communication, device management, data collection and analyses, scalability, and security, which control the design and evolution [1,2].

Implementing a stable model of this architecture becomes a reference architecture model. It is utilized and recommended to derive from a specified and concrete architecture, playing an essential role in the system of an application area, describing the model’s structure, and is a departure from developing tools. This implementation results in a framework that includes a minimal set of unifying concepts, axioms, and relationships responsible for understanding the interactions between the interties inside an environment [2].

Two main objectives needed to be achieved to guarantee the Industry 4.0 compliant integration: cross-layer structural connectivity and semantic interoperability between the component and the systems. Aim achievement requires the use of a reference architecture model, such as the Reference Architecture Model for Industry 4.0 (RAMI 4.0) or Industrial Internet Reference Architecture (IIRA) [3]. The communication layer becomes a critical piece of a reference architecture model [4]. However, the connection of smart devices, developed to work together, becomes an interoperability problem [5]. 

The IEEE defines interoperability as the “degree to which two or more systems, products or components can exchange information and use the information that has been exchanged” [6]. Another way to address an interoperability problem is by adopting standardizations [7]. Interoperability is divided into levels with different arrangements, depending on the author. The authors of [7,8] propose a division into five categories: device, network, syntactical, semantic, and platform. 

A syntactical level of interoperability uses data formats and data structures to exchange messages between systems. Each schema requires interfaces such as REST APIs, publish/subscribe, or client/server. The messages are encoded by the sender and decoded by the receiver; however, if the receiver decoding rules are incompatible with the ones from the sender, the message cannot be decoded [7].

Using the publish/subscribe pattern implemented in the Message Queue Message Protocol (MQTT) it is possible to achieve a syntactical level of interoperability. MQTT is an open messaging protocol developed to be lightweight, simple, and easy to implement [8]. With these characteristics, the MQTT is suitable for machine-to-machine (M2M) and IoT operations [9]. Additionally, the Hypertext Transfer Protocol (HTTP) can be used to communicate on the application layer inside an IoT context [10]. A middleware translates from one data format to another, promoting interoperability [11]. 

Semantic level interoperability enables different agents, services, and applications to exchange information with unambiguous meaning. It gives sense to the data presented inside a syntactic structure [12]. 

Frameworks (e.g., oneM2M and OPC UA) provide a semantic level of interoperability in Industry 4.0. The technical specifications are defined by the oneM2M, observing the needs of a standard M2M Service Layer [13]. The Open Platform Communications Unified Architecture (OPC UA) is a platform service-oriented to provide functionalities of an OPC Classic, being an implementation of the IEC 62541. This framework is platform-independent, secure, and extensible. OPC UA defines a series of specifications for the data transportation and an interface between the clients and the server [14].

Independent workgroups achieved a syntactical level of interoperability using a semantic gateway inside a service architecture. The proposed gateway provides the interoperability by translation between the message protocols (e.g., XMPP, CoAP, and MQTT) combined with W3Cs Semantic Sensor Network (SSN) ontology for semantic interoperability [15].

This paper implements the proposal of a syntactic interoperability between the IEEE 1451 family of standards and IEC 61499 inside a reference architecture model for validation purposes. The results found application in real-time data acquisition in industrial automation environments. The MQTT and HTTP protocols allowed to evaluate and compare the performance between a publish/subscribe method and a client/server method. Both IEEE 1451 and IEC 61499 implement the MQTT and HTTP protocols inside the application layer from ISO/OSI model. For a reference architecture model these protocols are inside the Transport layer. The experimental setup, installed in two different geographic locals, was used to collect the network’s data performance about latency estimation, packet size, packet loss, and retransmission time from a local network at UBI-PT and an external network at Miami-USA. A previous work achieved this level of interoperability and presented how to compare both standards and share properties to improve communication [16]. The semantic level is achieved by adopting a framework at a level of interoperability, resulting in a concrete implementation of a communication layer defined by a reference architecture model. The results showed that the adoption of publish/subscribe or client/server method depends on the level of reliability that the communication process requires. 

The remainder of this paper is structured as follows. Section 2 presents an overview of the reference architecture model, IEEE 1451 family of standards, IEC 61499 standard, and the related works. The proposed methodology is shown in Section 3. Section 4 describes and discusses the implementation of the experimental test setup. Section 5 presents the main discussion supported by the results and draws the conclusions.

## 2. Background

### 2.1. Industrial Internet Reference Architecture

The Industrial Internet Reference Architecture (IIRA), based on the ISO/IEC/IEEE 42010 [17], was developed in 2015 by the Industrial Internet Consortium (IIC) focused on IIoT systems. The IIRA is a guidance for the IIoT architectures, business leaders, and users of different levels, used to optimize and establish IIoT systems, enabling the conversion of OT (Operational Technology) and IT (Information Technology). For communication applications, the IIRA defines the Industrial Internet Architecture Framework (IIAF) that specifies the viewpoints and concerns during the development, documentation, and communication. 

The IIRA Volume G1: Reference Architecture [17] provides a generic description and representation for the high levels of common industry characteristics, features, and patterns. The Refence Architecture maximizes the value chain by implementing interoperability, mapping, and guiding the application technologies; besides, the IIRA is an open architecture for IIoT systems. The applicability’s scope of IIRA represents the incorporation of a generic architecture framework, as a reference architecture inside a real-world scenario, by the transformation and extension from an abstract architecture concept and models to a detailed architecture that can be utilized inside the industry [17]. 

**Figure 1:**
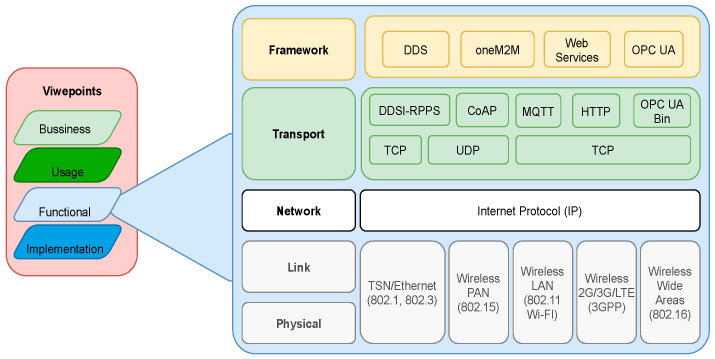
IIRA viewpoints and communication layer of IICF [18].

The definition of IIRA documentation employs business, usage, functional, and implementation viewpoints, as shown in Figure 1. The *Business viewpoint* identifies the stakeholders and their business vision, values, and objectives. The *Usage viewpoint* focuses on the system usage and represents the sequence of activities involving the users to deliver functionality to achieve the core of the system’s capabilities. The *Functional viewpoint* aims at the functional components, their structures, interrelation, interfaces and interactions, relation, and interactions of the systems with external elements in the environment, to support the usage and activities of the overall system. The *Implementation viewpoint* concentrates on the technologies used to implement the *Functional viewpoint*, has special attention to their communications schemes and life cycle procedures [17]. 

The *functional viewpoint* defines the architecture for the communication layer, functional capabilities, and structure for the IIoT infrastructure inside the IIRA. Five functional domains are defined: control, operations, information, application, and business. The main domain for the communication aspect relevant for this paper is the control domain that implements the control systems, composed of a collection of functions to implement the industrial control and automation systems [17]. 

The control domain comprises dedicated elements, such as sensing, actuation, communication, entity abstraction, modeling, asset management, and task executor. A collection of functions is defined for sensing, reading data from the transducer, applying logic rules, and writing data and controlling the signals over the physical system in the case of an actuator. A device localized inside this domain can interact with another placed in a geographically distributed arrangement.

Communication is responsible for connecting sensors, actuators, controllers, gateways, and other systems, using, for example, network architectures and Quality of Services (QoS). An entity abstraction enables the abstraction of sensors and actuators, controllers, and systems and expresses the relationships between them. Modeling comprises the states, conditions, and behavior of the systems. Asset management controls systems including onboarding systems, configuration, policy, system, software/firmware updates, and other lifecycle management operations. An ‘executor’ element executes the control logic, understanding the systems’ states, conditions, and behavior using the pre-defined control rules [17].

Another essential viewpoint for communication is the *Implementation viewpoint*. It defines a technical representation and general architecture for an IIoT system, technologies, and the requirement for the system components (e.g., interfaces, protocols, behaviors, and properties). The *Implementation viewpoint* implements the activities and functions described in the *Functional viewpoint*. It defines the topology used, technical description of the components, implementation map of actions identified in the usage viewpoint to the active components and its implementation [17].

### 2.2. IEEE 1451 Family of Standards

IEEE Instrumentation and Measurement Society supported the development of the IEEE 1451 standard to meet industry requirements. The standard provides a standard interface for smart transducers, ensuring the access of smart transducers through a communication network to support the data exchange between the network elements, allowing the manufacturers to build an interoperable system. The standard defines the following capacities to the smart transducers: identification, description, diagnosis, calibration, location, time, data processing, reasoning, data fusion, alert notification, data formats, and communication protocols [19]. 

The IEEE 1451 organizes itself as a family of standards. The IEEE 1451.0 defines a common set of commands and functionalities that provide smart transducers with access to the network and defines the Transducer Interface Module (TIM) that integrates with sensors and actuators. The TIMs perform signal conditioning tasks, converting signal domains from analogue to digital and digital to analogue. The Transducer Electronic Data Sheet (TEDS) describes TIM’s internal structure [20]. Several TEDS are embedded into the smart transducer, storing information about it, such as manufacturing identification, calibration, measurement, and accuracy [19]. 

The middleware communication between an application or network to the TIM is defined by the IEEE 1451.1 Network Capable Network Processor (NCAP). The NCAP manages communications over the user network using one of the following protocols: TCP/UDP, HTTP, XMPP, SNMP, and MQTT [21]. The NCAP also defines the following services: discovery of new a TIM, notification of events detected by sensors, and transducers management. 

Internally the NCAP is organized by classes of objects with a network-neutral interface. It is composed of blocks, services, and components for communicating with the transducers [21]. The NCAP Block enables software interfaces and supports network communication and system configuration. Base Transducer Block interfaces between the transducer and application functions. The Function Block is responsible for the encapsulation of the application-specific functionalities [21]. The Function Block encapsulates the application-specific functionalities [21] divided into physical and logical views. The physical view deals with physical components (sensors and actuators) that compound the smart transducers. A smart transducer interfaces with the users’ network, using an adequate microprocessor or a controller. The logical view deals with logical components grouped in an application to support the NCAP components (operating system, network protocol, and transducer’s firmware) [21]. 

The remaining IEEE 1451 family of standards is composed of the IEEE 1451.5 standard that defines the wireless communication between the NCAP and the TIM by one of the following protocols: Wi-Fi, 6LoWPAN, Zigbee, and Bluetooth [22]. At the same time, the IEEE 1451.2 standard specifies wired connections.

The IEEE 1451.0 defines an Application Programming Interface (API) using the HTTP protocol to ensure communication with the smart sensors. The API allows to request data from a sensor and send commands to an actuator [20]. Whereas, the IEEE 1451.1 species the client/server and publish/subscribe patterns ensure the interoperability between the smart transducer with the network. This family of standards addressed the characteristics to meet Industry 4.0 requirements [23].

### 2.3. IEC 61499 Standard

The IEC 61499 Function Block (FB) was proposed in 2005 by the Industrial-Process Measurement and Control Systems (IPMCS). This standard adopts the Function Block employed in the IEC 61131 standard for Programmable Logic Controller (P.L.C.) development [24]. To overpass the lack of flexibility and reusability, the IEC 61131 was modified and became the building block for the IEC 61499. Furthermore, the Distributed Control Systems (DCS) adopted it also. The IEC 61499 is hardware-independent from the user’s application to promote interoperability and reconfigurability between the devices. The system inside of an IPMCS is composed of a collection of IEC 61499 Devices interconnected by a network [25].

An IEC 61499 device is composed of at least an interface. The interfaces are divided into Process and Communication. Process Interface maps the physical entities and research, whereas the Communication Interface connects the resources for information exchange using a network. An IEC 61499 Device can be composed, or not, by resources or FBs. A Resource represents the functional unit inside a device, self-controlling its operation behavior. A Resource receives data/events from other resources or physical devices through a service interface. An Application can be a FB network or sub-applications connected by data and event connections. A FB is a fundamental building block for the IEC 61499, composed of input and output data and events, controlled by an Execution Control (EC) in the head and one or more algorithms in the body. When an input event occurs, the algorithm executes using the input data. After processing, an output event is generated.

The IEC 61499 is composed of the reaming standards: the IEC 61499-1 defines the architectural model for a distributed system, the IEC 61499-2 presents the requirements for software tools to support the systems, the IEC 61499-3 specifies the programming languages, and the IEC 61499-4 specifies the development rules for the compliance profiles [26].

### 2.4. Related Works

There are distinct methods (e.g., middleware, translators, mapping) to promote interoperability between two different standards inside an IIoT and I4.0 environment and implement a reference architecture model. 

Saito and Nishi developed a conversion method that translates messages between the MQTT, CoAP, XMPP, and SMTP using the IEEE 1451 standards [27]. Cruz et al. developed a systematic review about middleware used inside the IoT and proposed a new reference architecture for the IoT environment [11]. The author in [28] presented a conceptual data layer model that enables interoperability across domains, organizations, and enterprises focused on the Internet of Production (IoP). A syntactical level of interoperability was achieved using the IEEE 1451 and IEC 61499 in [16].

Roffia et al. developed using the publish/subscribe paradigm a semantic model inspired by the Smart-M3 concept at the information interoperability level [29]. A service orientated protocol that works on demand as a translator between the protocols CoAP, HTTP, and MQTT was developed by Derhamy et al. [30]. An architecture to allow interoperability between multiple platforms and standards was introduced by An et al. It promoted the interoperability between the FIWARE and oneM2M. A discussion on how to implement a semantic level of interoperability between the IEC 61499 and the IEEE 1451 is presented in [31]. 

Syntactic and semantic levels of interoperability were implemented in a framework described in [32]. This framework focuses on device discovery and interaction. Another middleware proposed by the author in [10], named SymbIoT, was developed to achieve syntactic and semantic levels of interoperability.

A comparison between MQTT and HTTP for the communication inside the IoT was developed by Wukkadada et al. [33] and by Yokomi and Sasaki [34]. Naik developed a paper comparing the protocols of the MQTT, CoAP, AMQP, and HTTP application layers [35]. 

The authors in [36] presented a survey about the reference architecture model for Industry 4.0 (RAMI 4.0) supported by a case study implementation of the RAMI 4.0 in a test scenario.

## 3. Methodology

**Figure 2:**
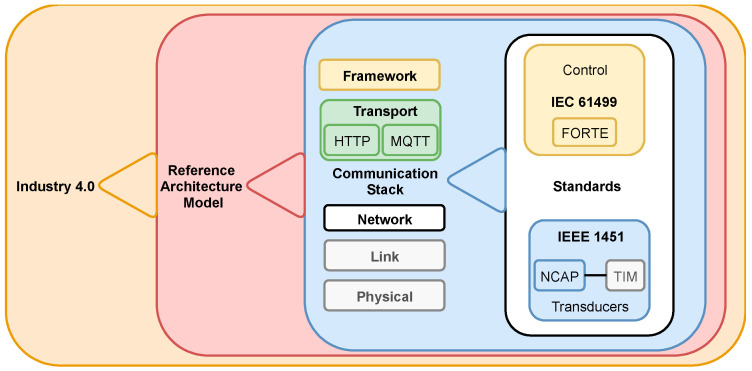
Encapsulation of an Industry 4.0 communication layer.

This section presents the methodology used to acquire data from a sensor. The syntactical interoperability between two protocols enables the message to go through the communication layer shown inside a reference architecture model. Figure 2 illustrates the concepts, standards, and communication layer.

Inside the functional viewpoint presented in the IIRA reference architecture model is the communication layer from the physical to the framework layer [18]. 

Both Standards IEEE 1451 and IEC 61499 support the MQTT and HTTP protocols at the Transport level of a reference model and the Application level of the ISO/OSI communication stack. 

The IEEE 1451 family of standards own the characteristics that meet the Industry 4.0 requirements [23]. The IEEE 1451.1 (NCAP) allows syntactic interoperability with other standards, as shown in [12,37]. The IEEE 1451.1-6 (under development) specifies communication by the MQTT protocols, whereas the IEEE 1451.1-2 specifies the connection by the client/server method [21]. A framework is necessary to achieve semantic interoperability.

The IEC 61499 has an FB that enables communication using the OPC UA framework allowing all the structures required for a reference architecture model for communication as presented in Figure 2, promoting data to the framework, such as OPC UA and oneM2M, also to client applications.

One of the essential aspects of the IEC 61499 function block is the ability to allow developers to pay attention to the application side more, rather than focusing on the hardware. This particular property is achieved by adopting the Service Interface Function Blocks (SIFBs). The publish/subscribe and client/server methods are particular types of SIFB called Communication Interface Function Blocks (CIFBs), proposed for unidirectional and bidirectional communication, respectively. The hardware vendors provide the necessary communication functionalities, preventing developers from building these interfaces. Both MQTT and HTTP applications use publish/subscribe and client/server methods available in 4diac. 

### Case Study for Validation

The IEC 61499 has FB that enables communication using the OPC UA framework. The IEC 61499 offers all the structures required by a reference architecture model to communicate. Figure 2 shows the encapsulation of an Industry 4.0 Communication layer.

The case study uses a car factory painting environment to test the communication and interoperability between the IEEE 1451 family of standards and the IEC 61499 standard. The quality of painting work depends on room temperature, and it is necessary to acquire temperature values from the painting sector and send them to the project partners. 

**Figure 3:**
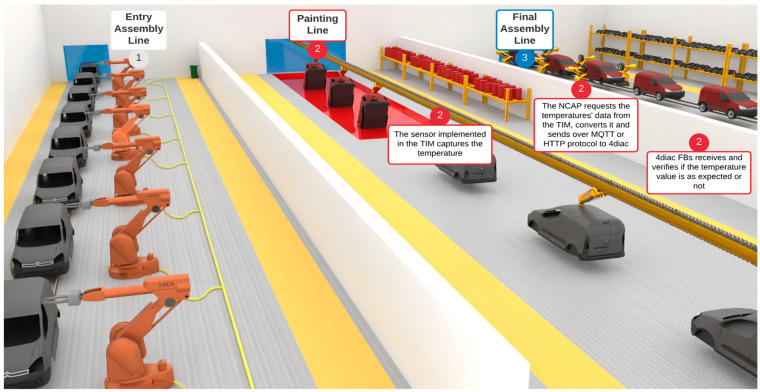
Case study representing a car painting line in a production plant.

The acquired data allows precise painting quality for the next step in the production chain, located in another part of the world, for example, in the USA. The user’s case scenario is shown in Figure 3.

The IEEE 1451 standard interfaces with the transducers, getting data from a temperature sensor connected to a TIM. At the same time, the IEC 61499 standard controls the procedure that requests and receives the data. The case study objectives are:

- to combine the IEC 61499 and the IEEE 1451 and achieve a syntactical level of interoperability;
- to allow both standards to manage data in the reference architecture model. This case uses the IIRA;
- to compare the publish/subscribe and client/server methods, highlighting the advantages and disadvantages of each one.

The IEC 61499 supports OPC UA in the framework layer, covering all the layers present inside the communication layer of the IIRA, enabling the IEC 61499 to send data for the upper layer of the communication present in IIRA, and finishing at the business layer. 

## 4. Implementation Evaluation

This section presents the testbench that implements the scenarios to test, followed by the test steps. Test scenario 1 uses the MQTT protocol, and test scenario 2 uses the HTTP protocol. Two different situations are evaluated in each of the scenarios. In the first situation, the communication starts in Portugal, ordering the data acquisition in Portugal, and finishes in Portugal. In the second situation, the transmission begins in the USA for data acquisition in Portugal and is sent to the USA. 

### 4.1. Sensor Implementation

The sensor is implemented inside a TIM as defined by the IEEE 1451 standards. A state machine manages the internal operation of a TIM. The TIM replies to particular commands sent from the NCAP to the TIM at each state. There are three operating states: “TIM Initialization”, “TIM Active”, and “Sleep”. The TIM Active state is composed of three other states: “Transducer Initialization”, “Transducer Idle”, and “Transducer Operating”. There is a specific command for each one of these states, also, commands for all states and state groups. The “Reset” command is the unique command for all states; it can be triggered at any time and places the TIM on the “TIM initialization” state [20].

The TIM initialization process starts at TIM’s power-up or after the execution of a hardware reset. When the TIM starts, it goes to the “TIM Initialization State” to read all the TEDS implemented inside the TIM, placing all the information and configuration data in the TEDS at the RAM and enabling the “Common Commands to TIM and TransducerChannel”. After that, it finishes the initialization process.

When the TIM receives a command from the “Common Commands to TIM and TransducerChannel”, the TIM changes to the “TIM Active” state. These commands contain destination addresses to the TIM or a TransducerChannel inside it, command class, and function.

The TIM on the “Transducer Idle” state enables the reception of commands to change TIM’s operation defined previously inside the TEDSs and loaded to TIM’s RAM. The “Sampling Mode”, “Data Transmission Mode”, and “Buffered state” are some of the operating parameters that can be changed.

At the “Transducer Operating” state, there are four commands that can be sent to the TIM, these commands are: “Trigger”, “Trigger Abort”, “Read TransducerChannel date-set segment”, and “Write TransducerChannel data-set segment”. 

The Trigger command can be addressed to a transducer channel, a transducer channel proxy, a group of addresses, or to all the transducer channels inside the TIM. The “Trigger Abort” command ends when a command “Trigger Abort” is received, and the “Trigger” command ends.

When the TIM receives a “Read TransducerChannel data-set segment” command from the NCAP, the TIM verifies the “sampling mode” configured for the sensor. The sensor starts to acquire the values and sends the answer to the NCAP. The IEEE 1451 standards specify five “sample modes”: “Trigger-initiated”, “Free-running without pre-trigger”, “Free-running with pre-trigger”, “Continuous sampling”, and “Immediate operation”. A transducer configured with “Immediate operation” will immediately acquire values from the sensor and answer the “Read TransducerChannel data-set segment”. The reception of this will work as a trigger for the transducer.

The reception of a “Write TransducerChannel data-set segment” command from the NCAP starts the digital to analogue conversion process, making the value available to the actuator placing it on the transducer channel port.

**Figure 4:**
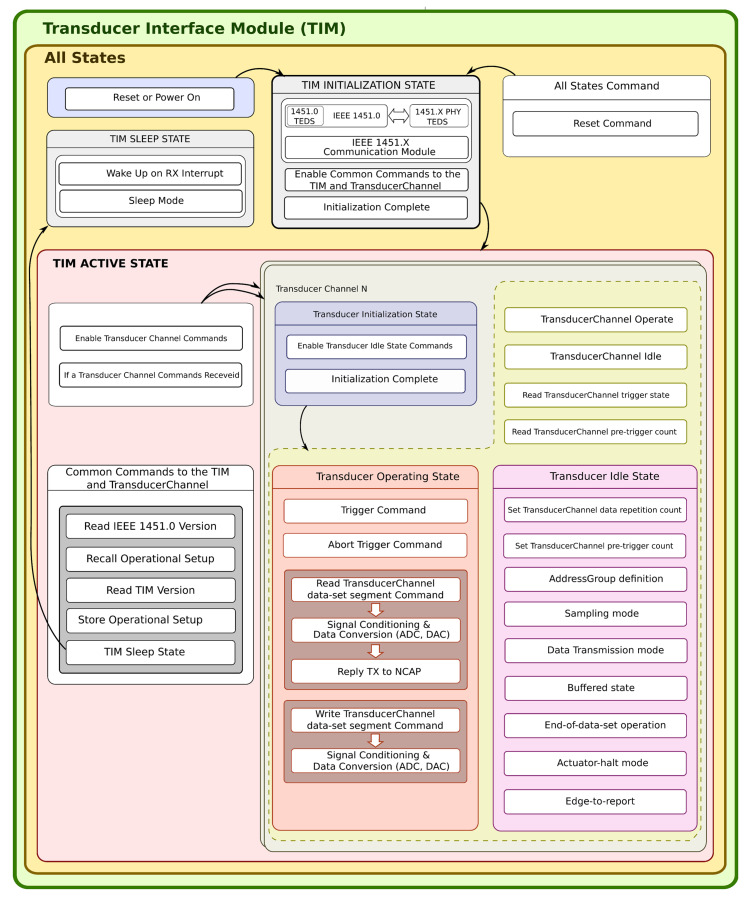
The state machine inside a TIM.

Figure 4 graphically shows the machine state and state transitions inside the TIM. For each state, the supported commands are also shown. 

A suite of tools developed to help build a TIM is available online [38]. A transducer channel connects the physical sensor to the TIM. A set of TEDS describes different aspects of the TIM. The TEDS are accessible by the user’s network through the NCAP connected to the TIM. The Meta TEDS provides the internal structure of the TIM. Each transducer channel has its own TransducerChannel TEDS that stores all the details about its configuration and operation. TEDS files were built using the “TIM TEDS Editor”. A project of a new TIM is built using the TEDS with the “TIM Project Editor”. The generated project is then imported by the Code Composer Studio [39] and, after functional coding, uploaded in an MSP430F5529 board. 

### 4.2. Equipment

The NCAP was coded using Python in a Raspberry Pi 3B+. The details about NCAP’s implementation are available in [40]. The NCAP receives the messages from the user’s application and interprets them as a command to send to the TIM using the UART connection. The implementation of the MQTT inside the NCAP uses Eclipse Paho MQTT [41]. For the HTTP implementation, the Flask project was used [42]. An API receives the request and answers with the data acquired by the sensor. The TIM was implemented using an MSP430F5529 board. Details about TIM’s implementation can be found in [38].

**Figure 5:**
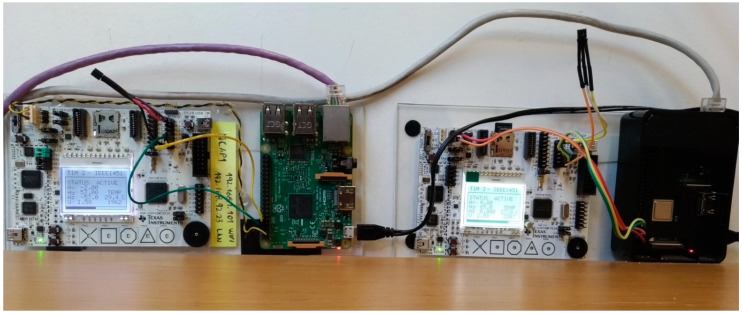
Experimental setup.

The 4diac was installed on a computer with a Windows 10, Core i7, 8 GB RAM, CPU 2.6 GHz, and 1TB HDD, and FORTE was installed in a Raspberry PI 3B at Miami University Ohio, USA. The second setup was installed in a computer with Windows 7 Ultimate, Intel Core i5-4440 CPU 3.10 GHz, 4 GB RAM, and 1 TB HDD, and FORTE program was installed in a Raspberry PI 3B+ at the University of Beira Interior (UBI), Portugal. The Wireshark program [43] was installed with the FORTE program inside the Raspberry devices. Figure 5 presents the experimental setup.

**Figure 6:**
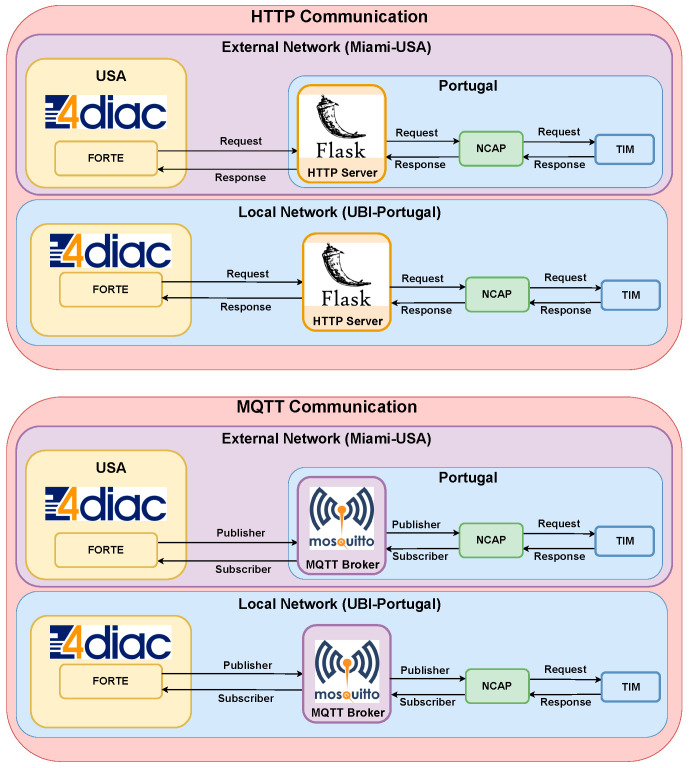
HTTP and MQTT communication.

The MQTT broker uses the Mosquitto broker [44] implementing the MQTT v3.1.1. The HTTP server uses the Flask project implementing HTTP v1, which was installed into a Raspberry Pi 3B+ at the University of Beira Interior, Portugal. This configuration is shown in Figure 6.

### 4.3. Proposed Test

A description of the communication process will be presented next. The main goal is to illustrate the differences between the client/server and publish/subscribe communication methods to connect the IEEE 1451 and IEC 61499 standards. An NCAP at UBI laboratory connects a TIM to the network. The TIM implements a temperature sensor that acquires the temperature value requested during the tests. The test steps to follow are:

1. Start the FORT Program in a Raspberry Pi 3B+;
2. Start Wireshark to capture the packets;
3. Start the 4diac program;
4. Using the MQTT protocol, the topic is subscripted to receive data, and starts to publish to the topic to request data every 10 min. Using the HTTP protocol, request the data using a URL every 10 min;
5. Perform the test for five days~40 thousand messages;
6. Stop Wireshark;
7. Analyze results.

**Figure 7:**
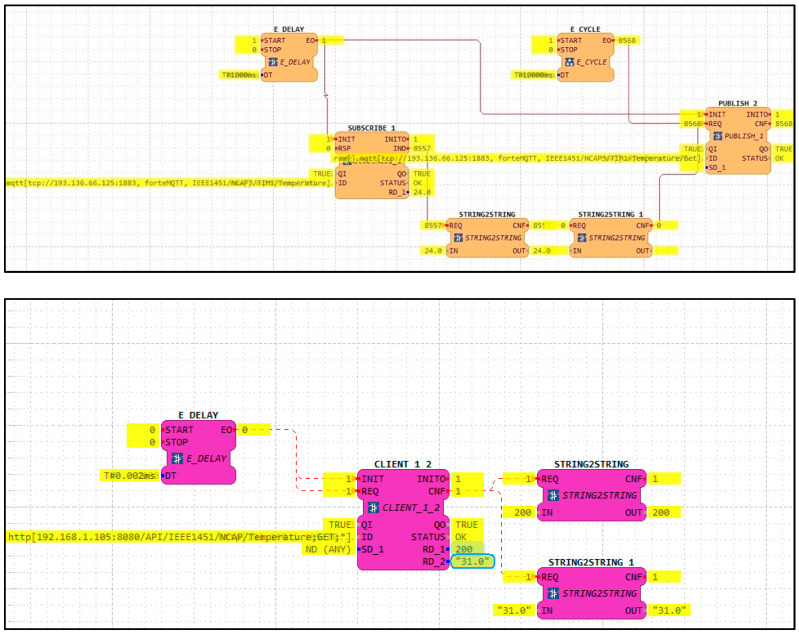
4diac MQTT and HTTP FBs.

Tests results will help understand how to take advantage of each protocol’s characteristics, depending on the application. The communication using the MQTT inside the IEC 61499 4diac program uses a Subscribe and a Publish FB. In comparison, the communication with the HTTP protocol uses a single Client FB. Figure 7 illustrates the implementation procedure.

### 4.4. Data Processed and Results

The Wireshark application captures packets during the communication process. The parameters analyzed in the communication process include latency observed in the communication process, packet loss, packet size, and initial retransmission time (iRTT) implemented in both protocols at the TCP level. 

**Figure 8:**
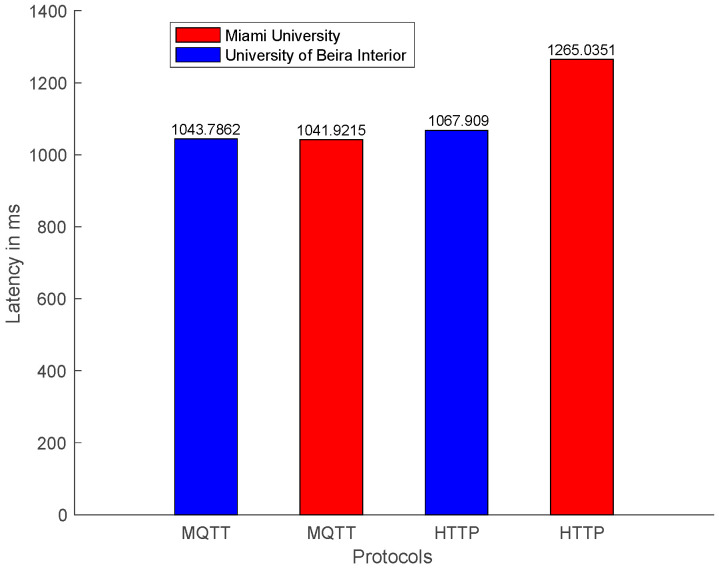
Mean of latency request and response.

The latency observed in the communication is shown in Figure 8. A message is published using the MQTT protocol, as shown in Figure 6. The NCAP publishes the answer to the broker. Both the publisher and subscriber used the QoS 0 supported by the 4diac program. QoS 1 and QoS 2 are not supported at this moment. For the HTTP protocol, a ‘*GET*’ command is sent using a client inside the 4diac program. Figure 8 shows that even publish/subscribe is not developed for request and answer methodology, and was faster than client/server request and response. 

The latency time for an MQTT subscriber was two times faster than HTTP. Both protocols have adequate performance, below 300 milliseconds that are expected for message exchange between continents [45]. 

**Figure 9:**
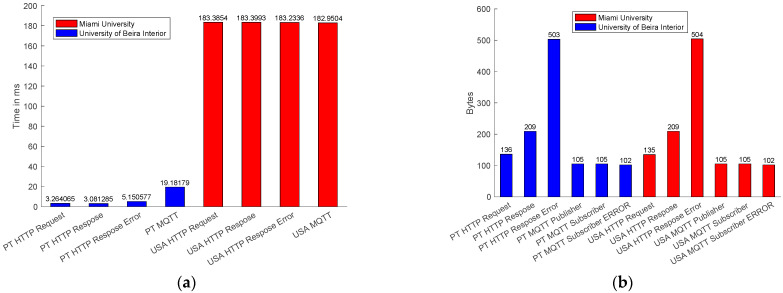
(**a**) Mean of iRTT delay; (**b**) packet size.

These results show that MQTT was faster even with an iRTT delay from the USA, worse than the local connection in Portugal, as shown in Figure 9a. 

The MQTT packet size was smaller than the HTTP package, as shown in Figure 9b. The MQTT packet size for the publisher and the subscriber is 105 bytes, whereas the HTTP packet size for request is 136 bytes and for response is 209 bytes. When the sensors get an error in data acquisition, the error packet size for MQTT was 102 bytes, and the error package for the HTTP was 504 bytes. 

**Figure 10:**
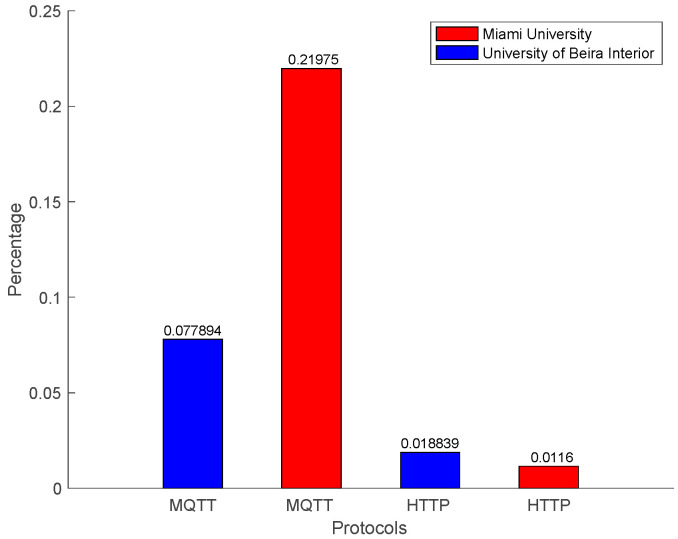
Packet loss.

MQTT was faster and used less bandwidth for communication. However, more packets were lost using the MQTT communication, as shown in Figure 10. 

The mean, standards deviation, median, minimum, and maximum values from the communication time from the communication that starts in Portugal and finishes in Portugal are presented in Table 1, and for communication that starts and ends in the USA are shown in Table 2. 

### 4.5. Evaluation and Discussion

Interoperability problems arrive from vendors offering different platforms to access data acquired in Industry 4.0 vendors, such as architectures, protocols, and semantics [46]. Two or more systems can exchange information and collaborate following diverse standards and architectures. Interoperability and reference architecture models can benefit from standardization, for example, the IIRA, as this work implemented. The IEEE 1451 family of standards was used to implement the device that acquires data, and the IEC 61499 was followed in the implementation of the control and monetarization system. Those standards are implemented inside the IIRA Function layer. Addressing interoperability was pursued using two paradigms: publish/subscribe and client/server.

For Industry 4.0, this paper studied characteristics such as the acquisition of data in real-time, communication, and monitoring. Two experimental setups were built and connected to a local network located at UBI, Portugal and an external network located at Miami, USA. Both universities had identical implementations for publish/subscribe and client/server methods. The IEEE 1451 family of standards and the IEC 61499 standard promote a syntactical level of interoperability using the application protocols MQTT and HTTP.

Latencies of data requests with origin in local and external networks using the MQTT and HTTP protocols are compared in Figure 8. Latency measures the elapsed time that goes from a MQTT publisher to publish a request to the corresponding topic inside the MQTT broker to the receiving of the requested information by the MQTT subscriber. Whereas, with the HTTP protocol, the latency is measured from the HTTP client sending the request to the HTTP server answer. For both cases, the IEC 61499 FORTE is the agent that always sends the requests. Data is collected by Wireshark and processed using MATLAB. From Figure 8, it is possible to conclude that even for MQTT, the local publisher/subscriber machine at UBI and MQTT external publisher/subscriber at Miami have similar answering times. It should be noticed that the sensor requires at least one second to acquire the temperature. The latency measured using the MQTT protocol for the local network was 43 ms and for the external network was 41 ms.

In contrast, retrieving data from the HTTP server, located at UBI, from a HTTP client located in an external network requires more time, precisely 265 ms. In comparison, it takes less time to retrieve the data for a client located in the local network. In [47] the author estimates the latency for an IIoT network in the same continent as 50 ms and between different continents as 300 ms. Both protocols, in local or external networks, had an excellent performance. The author in [48] establishes the latency for scale reading as 100 ms, making the MQTT and HTTP protocols, in a local network, adequate for Industry 4.0 reading data. In comparison, external requests to the HTTP server located at UBI takes longer to receive the data.

The communication packet size is compared in Figure 9b. It can be observed that they have similar sizes in the local and external networks. It is also possible to observe that a HTTP packet is almost twice as large as a MQTT packet, resulting in lower communication performance, as presented in Figure 8. However, when a sensor generates an error, the packet size for HTTP is bigger than the MQTT package because the HTTP server sends the page error, whereas the MQTT subscribers receive an empty message. In [48] the author requires a packet size of 512 bytes for scale reading systems, and the results present a packet size lower than the requirement. 

Packet losses during the communication are shown in Figure 10. One of the main characteristics of a real-time industrial environment is communication reliability. The MQTT protocol is faster and lighter. However, both in local and external networks, packet loss is higher when compared with the HTTP protocol. The HTTP performance in external networks is the best, similar to local networks. Figure 10 concludes that the HTTP protocol is more reliable at the moment with the implementation of IEC 61499 for measurement and control, whereas the MQTT is fast and lighter and can be used for an industrial application that does not need high reliability. 

Some limitations can be identified in the MQTT protocols since it has three Qualities of Service (QoS). The IEC 61499 FORTE only implements the QoS 0 with no confirmation message. In the future, if FORTE implements QoS 1 and QoS 2, the reliability of MQTT can increase. For the author in [48], the reliability for scale reading is 99%, which is achieved by the HTTP local and external networks. Whereas, the MQTT local network had a reliability of 93% and MQTT external network had 79%.

The better protocol solution when reliability is not the focus is the MQTT, which is faster, lighter, and consumes less bandwidth. The HTTP protocol is better for reliable communication. The MQTT protocol opens a connection and sends all the messages inside of it. In contrast, HTTP opens a connection for every request. However, the HTTP can wait for the request for longer periods of time, as presented in the maximum latency time in an external network, as presented in Table 2, that affects the mean latency network.

Both protocols can be used for the IIRA communication layer and promote a syntactical level of interoperability between the IEEE 1451 and IEC 61499. 

## 5. Conclusions

Communication has an essential role inside the Industry 4.0 environment. The reference architecture models were developed to specify how the communication needs to occur. The IIRA inside of this functional viewpoint presents the communication layer. For the transport layer of reference architecture models, MQTT and HTTP can be used. From the ISO/OSI, MQTT and HTTP are inside the Application layer. It enables the syntactical level of interoperability between the standards used inside of Industry 4.0. 

The IEEE 1451 family of standards provides what is necessary to work with transducers, from data acquisition to data conversion, sharing data with other infrastructures implemented with other standards, using one of the application protocols. Whereas the IEC 61499 can be used to control and manage the sensors, also, it provides a framework layer of IIRA by the implementation of OPC UA, promoting semantic interoperability. The joining of the IEEE 1451 and IEC 61499 complete the requirements for the IIRA communication layer. Consequently, both protocols can be used for communication inside an I4.0 environment. 

Both standards support the MQTT and HTTP protocols. The tests showed that MQTT protocol has a better performance when the message size and latency is the main point for communication. The HTTP has a better performance when a reliable connection is needed.

Some limitations can be identified in the setup. The IEEE 1451 family of standards and the IEC 61499 standard can only interoperate using the MQTT and HTTP. IEEE 1451.1 enables TCP/UDP, HTTP, WebServices, XMPP, and MQTT communication protocols. The IEC 61499 supports OPC UA, HTTP, ROS, MQTT, FMI, TSN, FBDK/IP, Modbus, OPC DA, OpenPOWERLINK, and Arrowhead. Even IEEE 1451 and IEC 61499 do not support other protocols that can commonly be used by Industry 4.0, such as CoAP and AMPQ.

Another limitation identified was that the MQTT developed inside the IEC 6499 FORTE only supports QoS 0. The IEEE 1451.1 MQTT supports QoS 0, QoS 1, and QoS 2. This limitation can influence message delivery once QoS 1 and QoS 2 have a confirmation message. Sending a message with QoS 1 from IEEE 1451.1 NCAP, the IEC 61499 only was allowed to receive QoS 0 messages from the broker. The same occurs for HTTP that the tools only support HTTP/1.0, and it interferes with the reliability of the message delivery.

Future research will focus on eliminating the framework layer of the IIRA and promoting semantic interoperability directly from the IEEE 1451 standard.

## Figures and Tables

**Table 1:** Communication that starts and ends in Portugal.

Starts in Portugal	Time in Milliseconds				
	Mean	Std. Deviation	Median	Minimum	Maximum
MQTT	1043.78	68.00	1035.85	0.0859	8030.33
MQTT With Error	1016.80	59.22	1016.80	23.273	1844.56
HTTP	1067.90	44.35	1062.10	108.566	4555.97
HTTP With Error	2056.31	193.09	2069.34	50.254	3299.96

**Table 2:** Communication that starts and ends in the USA.

Starts in the USA	Time in Milliseconds				
	Mean	Std. Deviation	Median	Minimum	Maximum
MQTT	1041.92	48.55	1038.60	0	4121.90
MQTT With Error	1024.01	42.07	1019.66	25.226	1560.68
HTTP	1265.03	693.42	1245.54	278.988	66107.82
HTTP With Error	2259.56	1099.64	2251.10	220.87	67149.64

## Abbreviations

The following abbreviations are used in this manuscript:

AMQP	Advanced Message Queuing Protocol
API	Application Programming Interface
CoAP	Constrained Application Protocol
CIFB	Communication Interface Function Blocks
CPS	Cyber-Physical Systems
CPPS	Cyber-Physical Production Systems
FB	Function Block
HTTP	Hypertext Transfer Protocol
I4.0	Industry 4.0
IEC	International Electrotechnical Commission
IEEE	Institute of Electrical and Electronics Engineers
IIoT	Industrial Internet of Things
IIRA	Industrial Internet Reference Architecture
IoT	Internet of Things
iRTT	Initial Retransmission Time
ISO	International Organization for Standardization
IT	Information Technology
M2M	Machine to Machine
MQTT	Message Queue Telemetry Transport
ms	Milliseconds
NCAP	Network Capable Application Processor
OPC UA	Open Platform Communications Unified Architecture
OSI	Open Systems Interconnection
OT	Operational Technology
PLC	Programmable Logic Controller
PT	Portugal
QoS	Quality of Service
RAMI 4.0	Reference Architecture Model for Industry 4.0
SIFB	Service Interface Function Blocks
SMTP	Simple Mail Transfer Protocol
SSN	Semantic Sensor Network
TEDS	Transducer Electronic Data Sheet
TCP	Transmission Control Protocol
TIM	Transducer Interface Module
UART	Universal Asynchronous Receiver-Transmitter
UBI	University of Beira Interior
URL	Uniform Resource Locator
USA	United States of America
XMPP	Extensible Messaging and Presence Protocol
